# Clinical and radiographic outcomes of hybrid graft in patients with Modic changes undergoing transforaminal lumbar interbody fusion

**DOI:** 10.1186/s13018-021-02652-7

**Published:** 2021-08-11

**Authors:** Jiaxun Jiao, Jiaqi Li, Yun Luo, Wei Zhang

**Affiliations:** 1 Department of Spinal Surgery, Harrison International Peace Hospital, Hengshui, 053000, Hebei People’s Republic of China; 2 Department of Spinal Surgery, The Third Hospital of Hebei Medical University, Shijiazhuang, 050051 People’s Republic of China; 3 The Key Laboratory of Orthopedic Biomechanics of Hebei Province, The Third Hospital of Hebei Medical University, Shijiazhuang, 050051 People’s Republic of China

**Keywords:** Modic changes, Transforaminal lumbar interbody fusion, Autologous local bone graft, Allogeneic freeze-dried bone graft, Fusion, Outcomes

## Abstract

**Background:**

This retrospective study aimed to analyze the influence of Modic changes (MCs) on the clinical and radiographic outcomes of transforaminal lumbar interbody fusion with hybrid graft.

**Methods:**

Clinical data of 89 patients with Modic changes who underwent single-segment transforaminal lumbar interbody fusion between January 2015 and January 2019 at our institution were analyzed. Patients were divided into three groups: the MCs-0 group (no endplate changes; used as the control group), the MCs-1 group, and the MCs-2 group. Clinical and radiological parameters were compared between the three groups.

**Results:**

There were no significant between-group differences in age (*P* = 0.216), sex (*P* = 0.903), body mass index (*P* = 0.805), smoking (*P* = 0.722), diagnosis (*P* = 0.758), surgical level (*P* = 0.760), blood loss (*P* = 0.172), operative time (*P* = 0.236), or follow-up (*P* = 0.372). Serum C-reactive protein level and erythrocyte sedimentation rate in the MCs-1 and MCs-2 groups were significantly higher than those in the MCs-0 group on the third and seventh days (*P* < 0.05). Postoperative radiographic parameters were significantly higher than preoperative parameters in all 3 groups (*P* < 0.05). Visual analog scale scores for low back pain in the MCs-0 and MCs-2 groups were significantly different from those in the MCs-1 group (*P* < 0.05). However, there were no significant between-group differences with respect to Oswestry Disability Index scores or visual analog scale scores for leg pain.

**Conclusion:**

In this study, Modic changes had no impact on fusion rates and clinical outcomes of transforaminal lumbar interbody fusion with hybrid graft (autologous local bone graft plus allogeneic freeze-dried bone graft). However, MCs-1 increased the risk of cage subsidence and showed superior outcomes in terms of visual analog scale scores for low back pain.

## Background

Transforaminal lumbar interbody fusion (TLIF) is a widely used procedure for treatment of degenerative lumbar spine disease. During the surgery, implantation of an intervertebral cage and autologous bone graft help restore the intervertebral height and promote fusion [1–3]. Bone graft material is one of the main determinants of intervertebral fusion. The common sources of bone graft used for TLIF include autologous iliac graft, autologous local bone graft, and allograft. Each bone graft material has its advantages and disadvantages. The use of autologous iliac graft or local bone graft is associated with reasonable fusion rate and does not entail the risk of rejection; however, availability of a limited amount of bone graft and increase in the postoperative recovery time are disadvantages. The use of allograft does not entail the problems of limited autologous bone supply and invasive bone retrieval. However, the treatment cost is typically high, and allografts are prone to rejection, osteolysis, and resorption [4–6].

Cage subsidence (CS) is one of the complications that results in gradual loss of disc height (DH) and segmental lordosis (SL). Previous studies have suggested that CS is associated with osteoporosis, excessive distraction of the intervertebral space, and damage to the endplate [7–10]. Modic changes (MCs) in the lumbar endplate and subendplate bones on magnetic resonance imaging (MRI) images are classified into three types: MCs-1 is considered as the inflammatory phase or edema phase; MCs-2 is considered as the fatty phase or yellow marrow phase; MCs-3 is considered as the osteosclerosis phase. Previous studies have shown that MCs are one of the reasons for low back pain and are closely related to lumbar degeneration. There is no clear consensus as to whether MCs affect the fusion. Some studies showed that MCs inhibit the process of fusion, as fusion rate after posterior interbody fusion was lower in patients with MCs. In contrast, other studies have shown good fusion results in patients with MCs [11–15].

MCs reflect an inflammatory dysmyelopoiesis which can induce biological and/or microstructural changes in the subchondral trabecular bone and affect the stable contact between the graft and the endplate [16, 17]. It is not clear whether the bone graft material affects intervertebral fusion and increases the risk of CS, especially when MCs are present in the endplate. We hypothesized that MCs affect the mechanical strength or biological properties of the subchondral bone and affect the fusion rate, especially after use of allograft during the surgery. In this retrospective study, we aimed to analyze the influence of MCs on the clinical and radiographic outcomes of TLIF with hybrid graft.

## Methods

Clinical data of 89 patients who underwent single-segment TLIF at the Third Hospital of Hebei Medical University between January 2015 and January 2019 were retrospectively analyzed. This study was approved by the Institutional Review Board of the authors’ institution. Written informed consent was obtained from all patients, and all clinical procedures were carried out according to the principles in the Declaration of Helsinki. Inclusion criteria were (1) patients with lumbar disc herniation (LDH), lumbar spinal stenosis (LSS), or lumbar spondylolisthesis (LS) who had significant symptoms and showed no response to at least 3 months of standardized conservative treatment; (2) availability of complete clinical and imaging data (x-ray examination, MRI, and CT); (3) single-segment TLIF with a single polymer polyetheretherketone (PEEK) cage; and (4) postoperative follow-up > 1 year.

Exclusion criteria were (1) presence of fracture, tuberculosis, or tumor; (2) intraoperative endplate damage or immediate postoperative radiographs revealing fusion settling; (3) previous history of surgery or trauma; (4) combined congenital or developmental deformities; and (5) osteoporosis (bone mineral density (BMD) *T* value ≤ − 2.5).

**Fig. 1:**
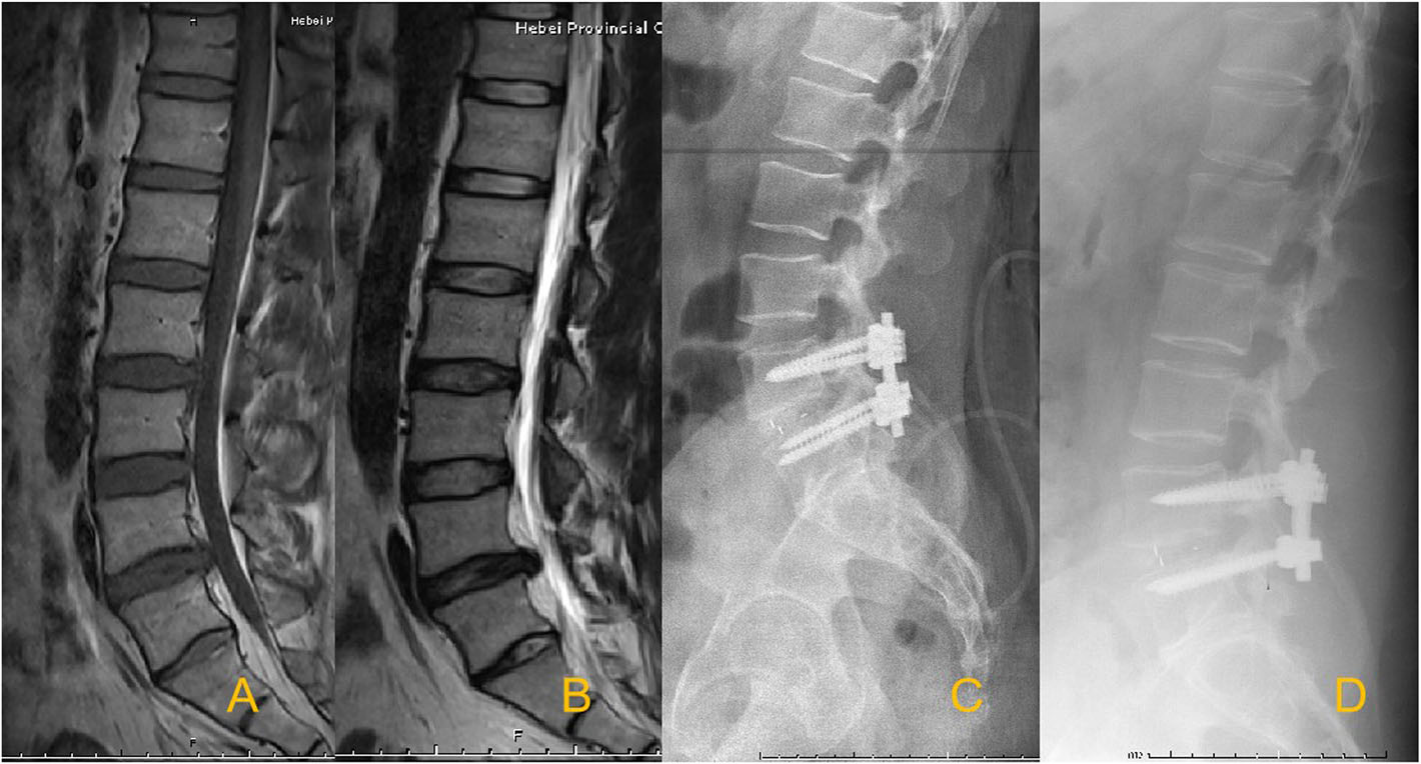
Sagittal T1-weighted (**A**) and T2-weighted (**B**) magnetic resonance images demonstrate L4−L5 lumbar disc herniation with MCs-0 signs. Postoperative radiographs (**C**) at the most recent follow-up confirming the solid fusion (**D**)

**Fig. 2:**
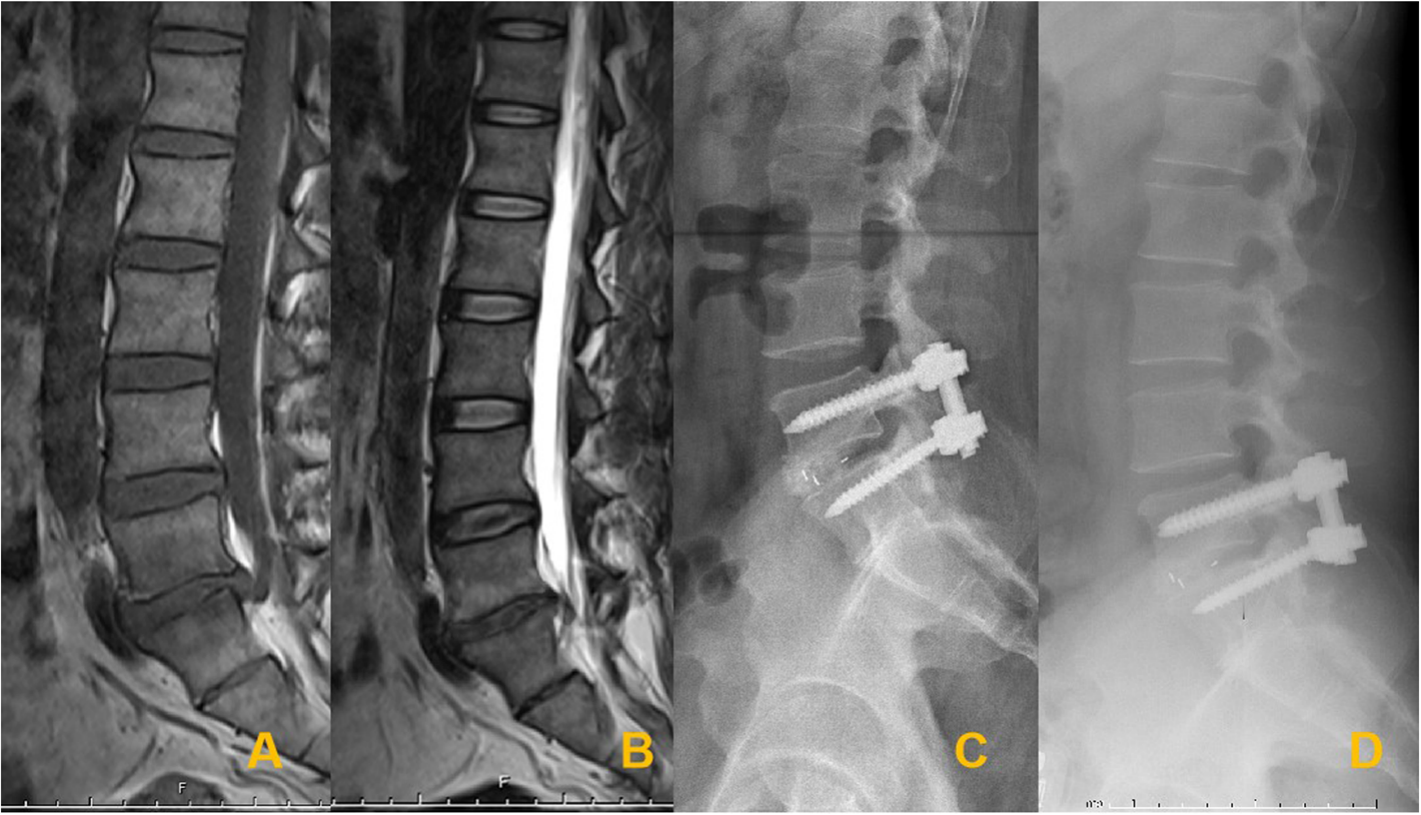
Sagittal T1-weighted (**A**) and T2-weighted (**B**) magnetic resonance images demonstrate L5−S1 lumbar disc herniation with MCs-1 signs. Postoperative radiographs (**C**) at the most recent follow-up confirming the solid fusion (**D**)

**Fig. 3:**
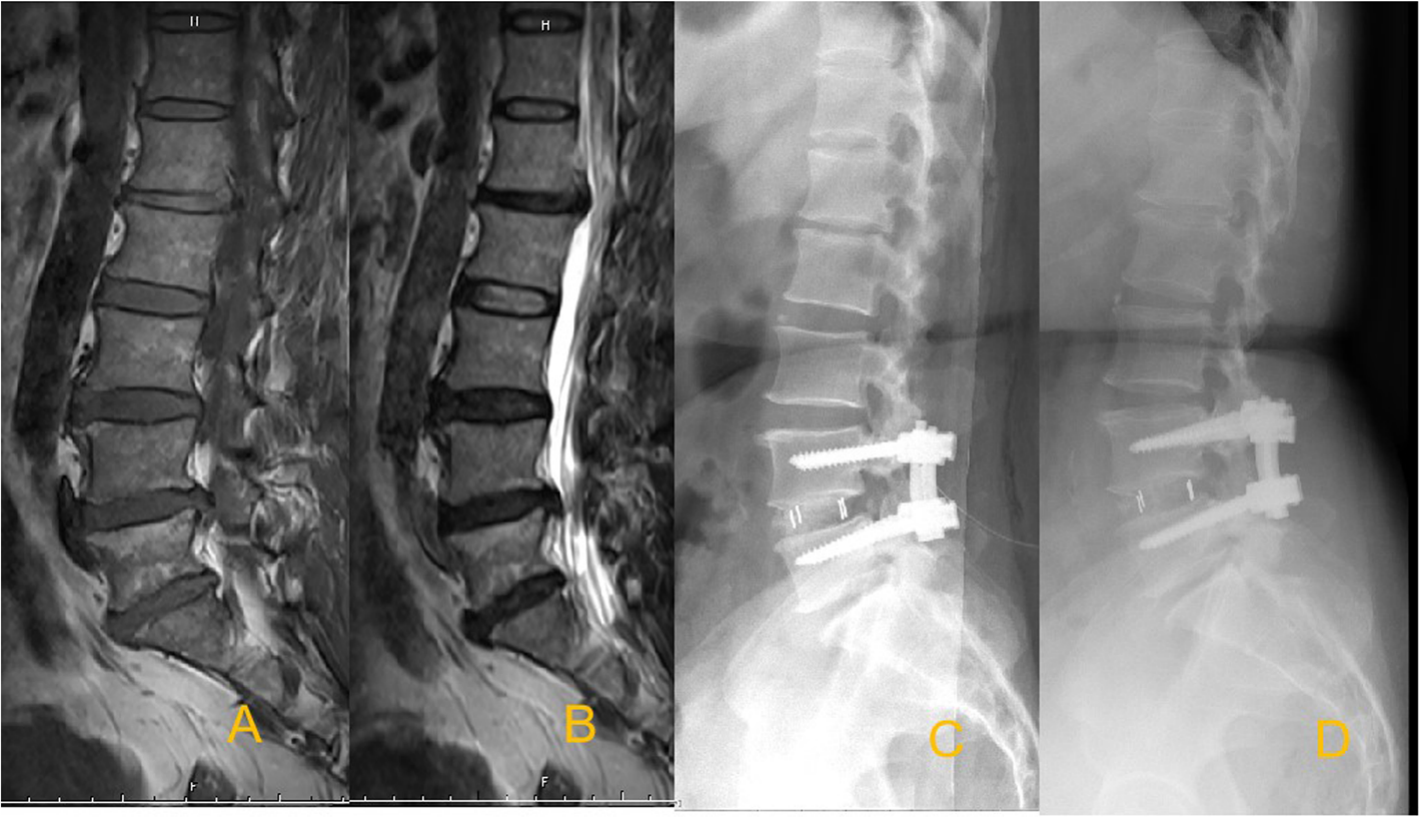
Sagittal T1-weighted (**A**) and T2-weighted (**B**) magnetic resonance images demonstrate L4−L5 lumbar disc herniation with MCs-2 signs. Postoperative radiographs (**C**) at the most recent follow-up confirming the solid fusion (**D**)

To assess the impact of MCs on the clinical outcomes of TLIF, patients were divided into three groups: the MCs-0 group (no endplate changes; used as the control group) (Fig. 1), the MCs-1 group (Fig. 2), and the MCs-2 group (Fig. 3). MCs-3 was not seen in the present study.

### Surgery

A conventional posterior median approach was used. The anatomical structures of the vertebral body and articular processes of the operated segment were exposed. The nail entry point was determined, and the pedicle screw was accurately placed. The more symptomatic side was used as the TLIF access side. Laminectomy and facetectomy were performed to expose the decompressed side. The intervertebral disc was dissected. The upper and lower endplates were also cleaned with a spatula, and the cartilage endplates were scraped to allow a small amount of blood to ooze from the bony endplates. After discectomy and endplate preparation, autologous local bone graft and allogeneic freeze-dried bone graft were filled into the intervertebral space. One PEEK cage of the appropriate size packed with autologous local bone graft was placed in the intervertebral area. The connecting rod was installed and held under pressure, followed by suture of the incision. Antibiotics were administered preoperatively and for 24 h postoperatively to prevent infection. The drainage was removed after the flow was less than 50 mL/24 h. During the period of bed rest, straight leg elevation functional exercises were performed, and the brace was used to protect the movement on the ground on the third day after surgery.

### Assessment parameters and measurements

Preoperative and postoperative data were recorded. For patients with longer follow-up, the most recent evaluation was included in the analysis. Preoperative MRI images were observed to determine the presence of MCs in the endplate. All MRI images were observed by two orthopedic surgeons who were blinded to the clinical outcomes. Disagreements, if any, were resolved by consensus.

**Fig. 4:**
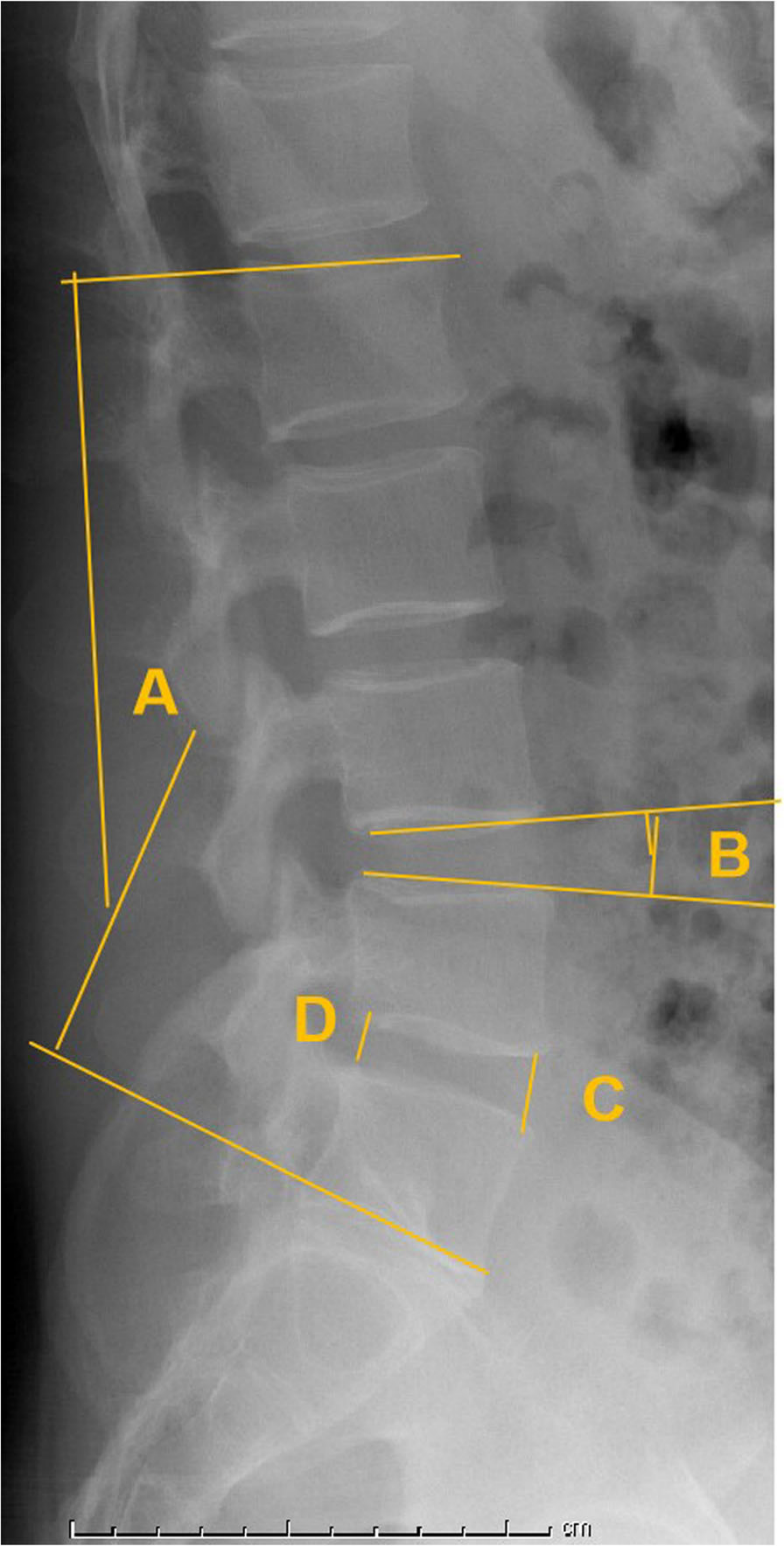
Lumbar lordosis (**A**): the angle between the upper endplate of L1 and the upper endplate of S1. Segmental lordosis (**B**): the angle subtended by the superior endplate line of the caudal vertebral body and the inferior endplate line of the cephalad vertebral body. Disc height: average of the anterior (**C**) and posterior (**D**) margins of the intervertebral space

**Fig. 5:**
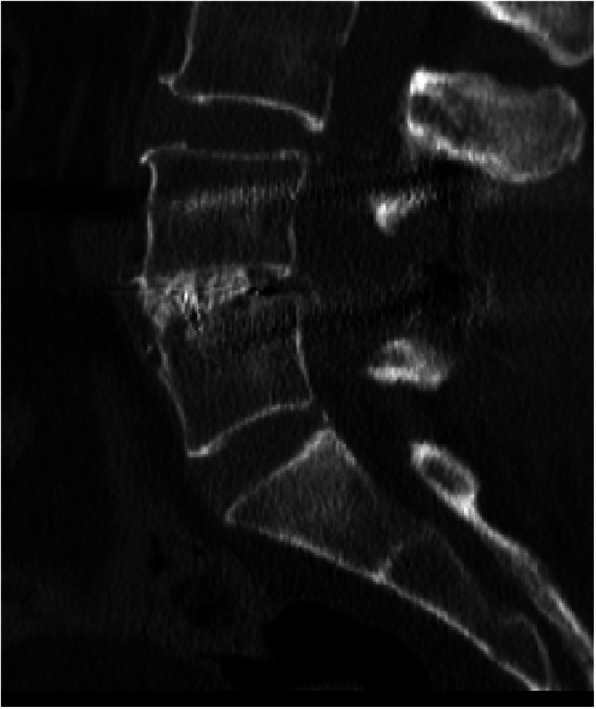
Solid fusion was considered to be achieved in the presence of bridging bone between the endplates of the cephalad and caudal vertebral bodies

The serum level of C-reactive protein (CRP) and erythrocyte sedimentation rate (ESR) were evaluated before surgery and on days 1, 3, 7, 15, and 21 postoperatively. The immunoturbidimetric method was used to determine the CRP level (< 10 mg/L). The modified Westergren method was used to measure the ESR (< 15 mm/h). The demographic data included age, sex, body mass index (BMI), surgical level, and complications. Radiographic parameters included lumbar lordosis (LL), which was measured as the angle between the upper endplate of L1 and the upper endplate of S1. Segmental lordosis (SL) was measured as the angle subtended by the superior endplate line of the caudal vertebral body and the inferior endplate line of the cephalad vertebral body. DH was defined as an average of the anterior and posterior margins of the intervertebral space (Fig. 4). CS was evaluated using postoperative images and described as sinking of the cage into the adjacent vertebral body by > 2 mm. Solid fusion was considered to be achieved in the presence of bridging bone between the endplates of the cephalad and caudal vertebral bodies (Fig. 5). Fusion was divided into four grades [18]. Patients with grades of 1 or 2 were considered to have achieved fusion (grade 1, fused with remodeling and trabeculae crossing vertebral endplate; grade 2, graft intact with no radiolucency but not fully remodeled; grade 3, graft intact, radiolucency present at top and bottom of the graft; and grade 4, collapse or resorption of the graft).

### Quality of life assessment

Questionnaires were administered to patients before surgery, at 3 months after surgery, and at the final follow-up to evaluate quality of life. The Oswestry Disability Index (ODI) scale was used to evaluate clinical efficacy, and visual analog scale (VAS) was used for assessment of low back pain and leg pain.

### Statistical methods

SPSS 21.0 software was used for statistical analyses. Continuous variables are presented as mean ± standard deviation. The paired *t* test was used to compare the preoperative and postoperative parameters. Repeated-measures analysis was used for CRP and ESR levels and Student−Newman−Keuls test was used for group-to-group comparisons. Comparison between the three different groups was conducted using one-way analysis of variance and post hoc Student−Newman−Keuls test. Two-sided *P* values < 0.05 were considered indicative of statistical significance.

## Results

There were 38 patients in the MCs-0 group, 20 patients in the MCs-1 group, and 31 patients in the MCs-2 group. All patients in this study experienced significant relief in back and leg pain. No serious complications, such as infection or nerve injury, were observed in any of the three groups. None of the patients required additional surgery because of recurrent or residual back and leg pain. There were no significant differences between the 3 groups with respect to preoperative demographic factors, including age (*P* = 0.216), sex (*P* = 0.903), BMI (*P* = 0.805), smoking (*P* = 0.722), diagnosis (*P* = 0.758), surgical level (*P* = 0.760), blood loss (*P* = 0.172), operative time (*P* = 0.236), or duration of follow-up (*P* = 0.372) (Table 1).

**Table 1:** Demographic data of patients in the 3 groups

	MCs-0 group	MCs-1 group	MCs-2 group	*P* value
Age (years)	55.6 ± 8.6	56.3 ± 11.2	58.3 ± 7.8	0.216
Gender (M/F)	18/20	9/11	13/18	0.903
BMI	25.7 ± 8.5	26.7 ± 7.8	26.3 ± 8.1	0.805
Smoking	13/25	9/11	12/19	0.722
Diagnosis				0.758
LDH	15	11	13	
LSS	13	5	12	
LS	10	4	6	
Surgical level				0.760
L3−L4	6	1	5	
L4−L5	20	12	16	
L5−S1	12	7	10	
Cage subsidence				
Subsidence	6	8	7	0.117
No subsidence	32	12	24	
Blood Loss (mL)	116.6 ± 21.1	120.8 ± 23.9	119.2 ± 19.5	0.172
Operative time (min)	79.6 ± 17.5	82.3 ± 20.8	80.5 ± 21.2	0.236
Follow up (month)	23.3 ± 3.8	22.4 ± 2.5	24.3 ± 5.6	0.372

All patients had achieved fusion (67 cases achieved grade I fusion, and 22 cases achieved grade II fusion). No cases of grade III or IV fusion were observed. CS was detected in 8 cases (40.0%) in the MCs-1 group, 7 cases (22.6%) in the MCs-2 group, and in 6 cases (15.8%) in the MCs-0 group. The percentage of patients in the MCs-1 group who experienced CS was significantly higher than that in the MCs-0 and MCs-2 groups (*P* < 0.05).

The CRP and ESR values increased in all three groups. The peak CRP level was reached on the third day, and the peak ESR level was reached on the seventh day after TLIF. The CRP and ESR levels decreased after reaching their peak values. The CRP and ESR levels in the MCs-1 and MCs-2 groups were significantly higher than those in the MCs-0 group on the third and seventh days (*P* < 0.05). However, there were no significant differences in CRP and ESR levels between the MCs-1 and MCs-2 groups after TLIF (*P* > 0.05) (Table 2).

**Table 2:** CRP and ESR values before surgery through postoperative day 21

	Preoperative	Postoperative				
		Day 1	Day 3	Day 7	Day 15	Day 21
CRP level, mg/L						
MCs-0 group	1.9 ± 0.8	20.3 ± 5.5*	58.2 ± 11.7*	23.8 ± 9.7*	8.6 ± 1.7*	2.3 ± 1.9
MCs-1 group	2.2 ± 1.1	26.3 ± 73*	66.3 ± 15.2*	30.6 ± 10.1*	10.7 ± 2.3*	5.3 ± 1.5
MCs-2 group	2.1 ± 0.9	24.8 ± 8.6*	62.9 ± 13.1*	27.3 ± 8.2*	9.3 ± 1.9*	3.6 ± 2.1
ESR, mm/h						
MCs-0 group	9.6 ± 2.5	19.5 ± 2.3*	39.6 ± 7.1*	46.6 ± 10.3*	23.5 ± 5.7*	11.3 ± 3.2
MCs-1 group	10.8 ± 3.1	22.5 ± 3.8*	45.1 ± 8.6*	56.1 ± 9.2*	33.6 ± 6.8*	15.6 ± 5.9
MCs-2 group	10.3 ± 2.6	20.3 ± 2.9*	43.9 ± 5.8*	52.8 ± 7.2*	28.6 ± 7.1*	13.2 ± 6.7

* Significant difference compared with the preoperative in each group.

The immediate postoperative DH, LL, and SL were significantly higher than those measured preoperatively in the 3 groups (*P* < 0.05). The DH increased significantly to 11.5 ± 1.9 mm, 11.3 ± 2.1 mm, and 11.6 ± 1.7 mm postoperatively and was maintained at 10.9 ± 2.1 mm, 9.7 ± 1.5 mm, and 9.8 ± 1.6 mm at the most recent follow-up in the MCs-0, MCs-1, and MCs-2 groups, respectively. The LL increased significantly to 54.3 ± 10.8°, 55.8 ± 11.3°, and 54.5 ± 13.6° postoperatively and was maintained at 53.5 ± 11.6°, 52.8 ± 13.3°, and 52.8 ± 15.1° at the most recent follow-up in the MCs-0, MCs-1, and MCs-2 groups, respectively. The SL increased significantly to 18.9 ± 6.3°, 18.2 ± 7.8°, and 18.5 ± 8.3° postoperatively, and was maintained at 18.1 ± 7.5°, 17.3 ± 6.7°, and 17.8 ± 8.7° at the most recent follow-up in the MCs-0, MCs-1, and MCs-2 groups, respectively. No significant between-group differences were observed with respect to the postoperative DH, LL, and SL or at the most recent follow-up (*P* > 0.05) (Table 3).

**Table 3:** Comparison of preoperative, postoperative and the final follow-up radiographic parameters in the 3 groups

	MCs-0 group	MCs-1 group	MCs-2 group	*P* value
Preoperative				
DH (mm)	9.7 ± 1.5	8.9 ± 2.1	8.7 ± 1.6	0.013
LL (°)	50.1 ± 9.5	49.6 ± 10.5	50.3 ± 10.5	0.631
SL (°)	16.3 ± 7.2	15.7 ± 6.2	15.8 ± 7.5	0.199
Postoperative				
DH (mm)	11.5 ± 1.9*	11.3 ± 2.1*	11.6 ± 1.7*	0.484
LL (°)	54.3 ± 10.8*	55.8 ± 11.3*	54.5 ± 13.6*	0.456
SL (°)	18.9 ± 6.3*	18.2 ± 7.8*	18.5 ± 8.3*	0.601
Final follow-up				
DH (mm)	10.9 ± 2.1*	9.7 ± 1.5*	9.8 ± 1.6*	0.027
LL (°)	53.5 ± 11.6*	52.8 ± 13.3*	52.8 ± 15.1*	0.693
SL (°)	18.1 ± 7.5*	17.3 ± 6.7*	17.8 ± 8.7*	0.436

* Significant difference compared with the preoperative in each group.

The ODI and VAS scores for low back pain and leg pain showed significant improvement after surgery in all 3 groups (*P* < 0.05). VAS scores for low back pain in both the MCs-0 and MCs-2 groups were significantly different from those in the MCs-1 group (*P* < 0.05). The VAS scores for low back pain in the MCs-2 group were slightly superior than those in the MCs-0 group; however, the between-group difference in this respect was not statistically significant (*P* > 0.05). The ODI and VAS scores for leg pain had significantly improved at the last follow-up (*P* < 0.05). However, the ODI and VAS scores for leg pain showed no significant difference among the 3 groups (*P* > 0.05) (Table 4).

**Table 4:** Comparison of preoperative, postoperative and final follow-up clinical outcomes in the 3 groups

	MCs-0 group	MCs-1 group	MCs-2 group	*P* value
ODI				
Preoperative	25.3 ± 8.8	26.7 ± 9.3	28.2 ± 8.5	0.257
Final follow-up	12.6 ± 10.3*	8.8 ± 10.3*	13.6 ± 9.8*	0.459
VAS for low back pain				
Preoperative	6.4 ± 2.7	7.6 ± 2.5	6.9 ± 3.2	0.153
Final follow-up	2.6 ± 1.9*	1.6 ± 1.5*	2.5 ± 1.7*	0.031
VAS for leg pain				
Preoperative	6.3 ± 2.8	6.2 ± 3.6	6.0 ± 2.2	0.582
Final follow-up	2.3 ± 2.1*	2.1 ± 1.9*	2.7 ± 1.9*	0.183

* Significant difference compared with the preoperative in each group.

## Discussion

Spinal fusion surgery is one of the effective methods for the treatment of lumbar degenerative diseases. The advances in surgical techniques have helped reduce the invasiveness of the posterior decompression approach [19–21]. The bone graft material is an important factor that affects spinal fusion. The ideal bone graft material should have the following characteristics: good biodegradability and biocompatibility, no or low immunogenicity, osteoconductivity and osteoinductivity, mechanical tolerance, ease of processing into various shapes and sizes, three-dimensional structure, and high porosity, which facilitates the placement and adhesion of various growth factors. The primary sources of bone graft for intervertebral fusion are autologous iliac bone graft, autologous local bone graft, and allogeneic bone graft [22–24].

The autologous local bone graft is removed during posterior decompression, which comprises of mostly cortical bone and partially cancellous bone. The autologous local bone graft enables faster healing and is less likely to collapse. However, it has a limited bone volume and lower bone quality (very little cancellous bone) compared with the autologous iliac bone graft (large bone volume and better bone quality) [25–27]. Autologous iliac bone graft is still the preferred bone graft material when the bone volume obtained from decompression cannot meet the demand for intervertebral bone grafting. It has a good pressure-bearing capacity, reasonable fusion rate, and no rejection reaction. However, it can prolong the operation and increase the recovery time and postoperative pain [28]. Allogeneic freeze-dried bone is made from fresh bone tissue after deep freezing, drying, and gamma radiation sterilization. Its processing, sterilization, and preservation techniques are gradually becoming mature and increasingly being applied. It is the most widely used bone graft material in China and overseas in recent years. The use of allogeneic freeze-dried bone graft precludes the problems of limited autologous bone supply and does not entail invasive bone extraction. Numerous studies have confirmed that homogeneous freeze-dried bone exhibits strong osteoconductivity and is a relatively ideal material or cell carrier for bone defect repair, in addition to retaining some of its biologically active osteoinductive components [29, 30]. However, it still has some shortcomings compared with autologous bone graft; these include the risk of immune rejection, inflammatory reactions, reduced osteoinductive activity, decreased mechanical strength after long-term preservation, high incidence of bone resorption, and poor loading capacity.

Postoperative CRP and ESR levels are routinely monitored to detect postoperative inflammation and infection. In this study, the CRP levels were elevated earlier than ESR values, peaked on the third day after surgery, and returned to normal levels around the fifteenth day. The ESR levels reached their peak on the seventh day and returned to normal around the twenty-first postoperative day. The CRP level was a more sensitive index compared with the ESR. However, the trends of CRP and ESR levels were similar in the three groups. The CRP and ESR levels in the MCs-1 and MCs-2 groups were significantly higher than those in the MCs-0 group. However, there were no significant differences between the MCs-1 and MCs-2 groups in this respect.

Previous studies have found no significant difference in the fusion rate achieved with the use of autologous iliac bone graft and autologous local bone graft for lumbar fusion surgery. However, no studies have compared the fusion rate in the context of use of autologous local bone graft and allogeneic freeze-dried bone graft. In previous studies, the amount of bone graft showed a positive correlation with the fusion rate of the bone graft [25, 27, 31]. Use of allogeneic freeze-dried bone as a supplement for autologous local bone graft has some theoretical advantages in that it provides a larger bone volume and a higher proportion of cancellous bone. In the present study, to avoid mechanical instability due to excessive bone removal by decompression, we performed expanded “windowing” decompression of the vertebral lamina. Therefore, the amount of bone graft during intervertebral fusion was often insufficient, and was supplemented with an allogeneic bone graft. Thus, allogeneic freeze-dried bone graft plus autologous local bone graft was used to ensure sufficient bone volume.

MCs-1 represent an active and inflammatory phase of the endplate. During the surgery, a large discectomy could result in removal of the inflamed endplate and induce various inflammatory factors, which may be the reason for the relief of low back pain. Subsequently, the lumbar stability was reestablished by internal fixation and bone graft fusion, thus alleviating further damage to the endplate. All patients in this study underwent TLIF. After scraping off the endplate, a PEEK cage of the appropriate size packed with autologous bone graft was placed in the intervertebral space. All the three groups achieved fusion grade 1 or 2 at the most recent follow-up with satisfactory results. Although there was a loss of intervertebral height at the last follow-up, it was still higher than the preoperative level and did not affect the quality of life, which may be due to a less degree of subsidence.

The risk factors for intervertebral infusion or CS include damage of endplate, osteoporosis, graft type, number of fused segments, and over distraction of intervertebral height. Kwon et al. conducted follow-up of 351 patients for 3 years and found that the clinical outcomes and bony fusion rates were significantly lower in patients with MCs [17]. Cao et al. demonstrated PLIF as a reliable treatment for patients with MCs and predominant low back pain [32]. In this study, there were no significant differences between the three groups in terms of operative time, intraoperative blood loss, or complications, indicating that the surgical difficulty of the three implant fusion modalities was comparable. In the present study, the incidence of subsidence was 40% in the MCs-1 group, which further confirmed that the endplate is closely related to CS and fusion. The CS rate in the MCs-1 group was significantly higher than that in the MCs-0 group or MCs-2 group. The CS rate in the MCs-2 group was slightly higher than that in the MCs-0 group, indicating that MCs affect fusion. The damage of endplate can reduce the load-bearing capacity of the endplate and cause CS. The microenvironmental alterations caused by MCs affect the nutrient supply to the endplate. This weakens the endplate strength. This explains the higher rate of CS in the MCs-1 group as compared with that in the MCs-0 and MCs-2 groups.

We believe that careful intraoperative manipulation is essential to achieve successful fusion and reduce CS. The authors recommend scraping the cartilage endplate to remove part of the lesion during surgery and cleaning the endplate so that it oozes a small amount of blood; this can promote blood supply in the bone graft area and facilitate fusion. Preserving the intact bony endplate and preventing damage to the endplate may help avoid settling of the fusion apparatus. To ensure the amount of bone graft, allograft bone can be used to promote fusion, and a suitable intervertebral cage can be selected to avoid over distracting the intervertebral height and cause damage to the endplate. On the other hand, to improve the longitudinal support of the intervertebral bone graft, large pieces of decompression bone were preserved as much as possible during decompression, especially in the early stage of decompression. The inferior articular process in the surgical space was removed by chiseling with a bone knife, and the whole piece was trimmed and implanted into the surgical space to provide sufficient longitudinal support and reduce the loss of distant correction. To achieve successful fusion, the cartilage should be removed from the surface without causing injury to the subchondral trabecular bone. Failure of the subchondral trabecular bone to support the compressive load between the intervertebral graft and vertebrae during graft incorporation may cause loss of the disc space restoration or graft subsidence/nonunion/failure [33–35].

### Limitations

Some limitations of this study should be considered while interpreting the results. First, we did not evaluate the status of the endplate during the surgery, which may influence the fusion. Some patients underwent rehabilitation exercises at other institutions, which could not be accurately controlled for in the analysis. Second, the small sample size and retrospective nature of the study made it difficult to manifest all types of MCs, and to exclude the possibility of selection bias. Due to the natural distribution characteristics of MCs, none of the patients in our cohort had MCs-3. Third, there was a lack of postoperative MRI data; thus, potential change in the type of MCs could not be determined. Furthermore, it is believed that no substitutable grafts can achieve excellent fusion rate, or exhibit biocompatibility and osteoconductivity comparable to that of autologous bone. Our results need to be further confirmed by long-term follow-up and a larger randomized controlled study.

## Conclusion

In this study, MCs had no impact on fusion rates and clinical outcomes after TLIF with the hybrid graft (autologous local bone graft and allogeneic freeze-dried bone graft). However, MCs-1 increased the risk of cage subsidence and showed superior outcomes in terms of low back pain.

## Data Availability

Not applicable

## Abbreviations

TLIF: Transforaminal lumbar interbody fusion
CS: Cage subsidence
DH: Disc height
SL: Segmental lordosis
MCs: Modic changes
MRI: Magnetic resonance imaging
LDH: Lumbar disc herniation
LSS: Lumbar spinal stenosis
LS: Lumbar spondylolisthesis
CRP: C-reactive protein
ESR: Erythrocyte sedimentation rate
ODI: Oswestry Disability Index
VAS: Visual analog scale
