# Correction to: Clinical and radiographic outcomes of hybrid graft in patients with Modic changes undergoing transforaminal lumbar interbody fusion

**DOI:** 10.1186/s13018-022-03198-y

**Published:** 2022-06-16

**Authors:** Jiaxun Jiao, Jiaqi Li, Yun Luo, Wei Zhang

**Affiliations:** 1 Department of Spinal Surgery, The Third Hospital of Hebei Medical University, Shijiazhuang, 050051 People’s Republic of China; 2 Department of Spinal Surgery, Harrison International Peace Hospital, 053000 Hebei, People’s Republic of China; 3 The Key Laboratory of Orthopedic Biomechanics of Hebei Province, The Third Hospital of Hebei Medical University, Shijiazhuang, 050051 People’s Republic of China

## Correction to: Journal of Orthopaedic Surgery and Research (2021) 16:486 10.1186/s13018-021-02652-7

Following publication of the original article [1], the order of the first author’s affiliations was incorrect. The corrected order of the authors’ affiliations is as below:

Jiaxun Jiao^1,2,3^, Jiaqi Li^1,3^, Yun Luo^1,3^, Wei Zhang^1,3^*

^1^Department of Spinal Surgery, The Third Hospital of Hebei Medical University, Shijiazhuang 050051, People’s Republic of China.

^2^Department of Spinal Surgery, Harrison International Peace Hospital, Hengshui 053000, Hebei, People’s Republic of China.

^3^The Key Laboratory of Orthopedic Biomechanics of Hebei Province, The Third Hospital of Hebei Medical University, Shijiazhuang 050051, People’s Republic of China.

All authors agree to these corrections and apologize for the inconvenience caused.

The original article has been revised.
